# Erythropoietin Amplifies Stroke-Induced Oligodendrogenesis in the Rat

**DOI:** 10.1371/journal.pone.0011016

**Published:** 2010-06-11

**Authors:** Li Zhang, Michael Chopp, Rui Lan Zhang, Lei Wang, Jing Zhang, Ying Wang, Yier Toh, Manoranjan Santra, Mei Lu, Zheng Gang Zhang

**Affiliations:** 1 Department of Neurology, Henry Ford Hospital, Detroit, Michigan, United States of America; 2 Department of Physics, Oakland University, Rochester, Michigan, United States of America; 3 Department of Biostatistics and Research Epidemiology, Henry Ford Hospital, Detroit, Michigan, United States of America; Brigham and Women's Hospital, Harvard Medical School, United States of America

## Abstract

**Background:**

Erythropoietin (EPO), a hematopoietic cytokine, enhances neurogenesis and angiogenesis during stroke recovery. In the present study, we examined the effect of EPO on oligodendrogenesis in a rat model of embolic focal cerebral ischemia.

**Methodology and Principal Findings:**

Recombinant human EPO (rhEPO) at a dose of 5,000 U/kg (n = 18) or saline (n = 18) was intraperitoneally administered daily for 7 days starting 24 h after stroke onset. Treatment with rhEPO augmented actively proliferating oligodendrocyte progenitor cells (OPCs) measured by NG2 immunoreactive cells within the peri-infarct white matter and the subventricular zone (SVZ), but did not protect against loss of myelinating oligodendrocytes measured by cyclic nucleotide phosphodiesterase (CNPase) positive cells 7 days after stroke. However, 28 and 42 days after stroke, treatment with rhEPO significantly increased myelinating oligodendrocytes and myelinated axons within the peri-infarct white matter. Using lentivirus to label subventricular zone (SVZ) neural progenitor cells, we found that in addition to the OPCs generated in the peri-infarct white matter, SVZ neural progenitor cells contributed to rhEPO-increased OPCs in the peri-infarct area. Using bromodeoxyuridine (BrdU) for birth-dating cells, we demonstrated that myelinating oligodendrocytes observed 28 days after stroke were derived from OPCs. Furthermore, rhEPO significantly improved neurological outcome 6 weeks after stroke. In vitro, rhEPO increased differentiation of adult SVZ neural progenitor cells into oligodendrocytes and enhanced immature oligodendrocyte cell proliferation.

**Conclusions:**

Our in vivo and in vitro data indicate that EPO amplifies stroke-induced oligodendrogenesis that could facilitate axonal re-myelination and lead to functional recovery after stroke.

## Introduction

Oligodendrocytes are the myelin-forming glial cells in the adult brain and are highly vulnerable to ischemic insult [1], [2], [3]. Garcia and his colleagues demonstrate that as early as 30 minutes after middle cerebral artery occlusion (MCAo), animals exhibit swelling of oligodendrocytes followed by white matter injury [2]. Regeneration of mature oligodendrocytes has been observed in the peri-infarct areas after stroke [4], [5]. Mature oligodendrocytes in adult rodent brain are derived from non-myelinating oligodendrocyte progenitor cells (OPCs) that are present in the corpus callosum and the striatum [6], [7] Neural progenitor cells in the subventricular zone (SVZ) of the lateral ventricles also give rise to OPCs that disperse throughout the corpus callosum and striatum [8]. Mature oligodendrocytes in the ischemic boundary are likely generated from the OPCs because mature oligodendrocytes are generally considered incapable of cell division [9], [10], [11]. There are a paucity of studies that have investigated regeneration of oligodendrocytes in the ischemic brain during long-term stroke recovery [12]. Understanding of how OPCs and new oligodendrocytes contribute to ischemic repair is important for the development of therapies aimed at facilitating generation of mature oligodendrocytes that could promote remyelination leading to functional improvement after stroke.

Erythropoietin (EPO), a hematopoietic cytokine, facilitates oligodendrocyte maturation in vitro [13]. In experimental models of multiple sclerosis, spinal cord injury, cortical infarction, and neonatal hypoxia-ischemia, EPO treatment increases OPC proliferation and myelinating oligodendrocytes [14], [15], [16], [17]. In the current study, we investigated the effect of EPO on oliogdendrogenesis in a rat model of embolic stroke.

## Results

### The effect of EPO on functional outcome and infarct volume

**Figure 1:**
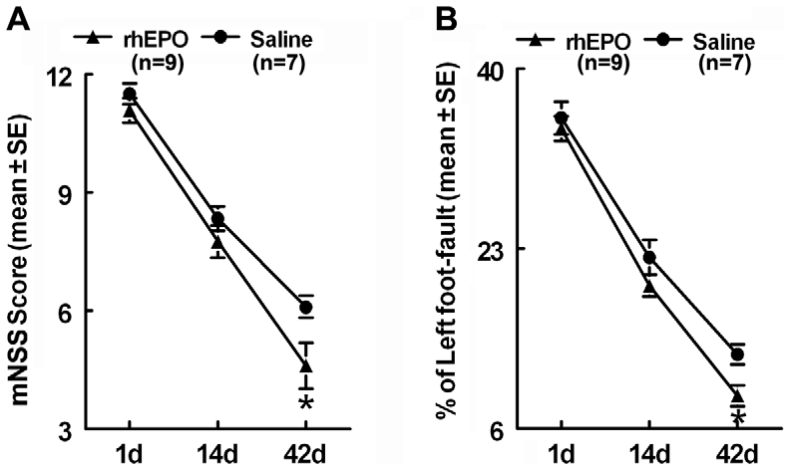
Neurological functional outcome. Panels A and B show the neurological functional outcome measured by mNSS (A) and foot-fault test (B) 1, 14, and 42 days after MCAo. Values are mean ± SE. *P<0.05 as compared with the saline-treated group.

To test the restorative effect of recombinant human EPO (rhEPO) on stroke, rhEPO (5,000 units/kg) was intraperitoneally administered daily for 7 days starting 24 h after stroke onset. Neurological deficits were examined before the treatment and 2 and 6 weeks after stroke by means of modified neurological severity score (mNSS) and foot-fault test. All rats exhibited severe deficits measured by mNSS and foot-fault test 24h after middle cerebral artery (MCA) occlusion and there were no significant differences among the groups (Fig. 1). Although rats treated with saline exhibited spontaneous improvements of behavioral outcomes, treatment with rhEPO significantly (*P*<0.05) improved neurological outcome compared with saline treated rats 6 weeks after stroke (Fig. 1), which is consistent with published studies [18], [19]. The ischemic lesion was not significantly (*P*>0.05) different between rats treated with saline (32.8±4.4% of the contralateral hemisphere) and rhEPO (34.4±5.6%).

### The effect of EPO on OPCs and myelinating oligodendrocytes in the ischemic brain

**Figure 2:**
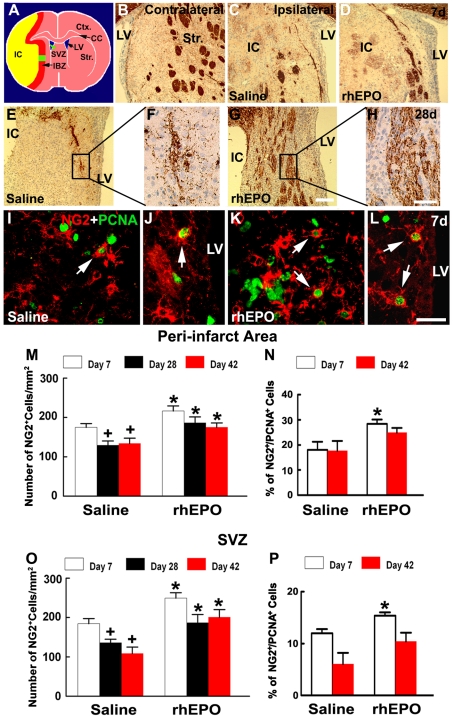
Effects of EPO on oligodendrocytes. Panel A is a schematic representation of a brain coronal section showing peri-infarct striatum (green box in A) and ipsilateral SVZ (green arrowhead in A) where all representative microscopic images were acquired. The yellow color represents ischemic core, and the red color indicates peri-infarct region. Panels B to H show CNPase immunoreactivity of representative rats treated with saline (B, C, E, F) and rhEPO (D, G, H), which were acquired from ipsilateral (C to H) and contralateral (B) striatum 7d (B to D) and 28d (E to H) after stroke. Panels F and H are high magnification images from boxed areas in panels E and G, respectively. Panels I to L show the double fluorescence immunostaining of NG2 (red) with PCNA (green), from representative rats treated with saline (I, J) and rhEPO (K, L), which were acquired from peri-infarct striatum (I, K) and SVZ (J, L). Panels M and O are the quantitative data of NG2 positive cells in peri-infarct corpus callosum and striatum (M), and ipsilateral SVZ (O). Panels N and P are the quantitative data of NG2 and PCNA double positive cells in peri-infarct corpus callosum and striatum (N), and ipsilateral SVZ (P). Values are mean ± SE. *P<0.05 vs saline. ^+^ *P*<0.05 vs the 7 day group. IBZ  =  ischemic boundary zone, CC  =  corpus callosum; Ctx  =  cortex; IC  =  ischemic core; LV  =  lateral ventricle; Str  =  striatum; SVZ  =  subventricular zone. Scale bars: 100 µm for panels B, C, D, E, and G, 50 µm for panels F and H. For 7 days, n = 4/saline and n = 4/rhEPO; for 28 days, n = 7/saline and n = 5/rhEPO; for 42 days, n =  7/saline and n = 9/rhEPO.

Aforementioned data suggest that the effect of EPO on improvement of functional outcome is not primarily resulted from the neuroprotective effect. To examine the effect of EPO on oligodendrocytes in the ischemic brain, we measured OPCs and myelinating oligodendrocytes 7, 28 and 42 days after embolic ischemia. OPCs were identified by NG2 positive cells, while myelinating oligodendrocytes were detected by cyclic nucleotide phosphodiesterase (CNPase) or myelin basic protein (MBP) immunoreactive cells [20]. CNPase immunostaining revealed that 7 days after MCAo, CNPase immunoreactivity was essentially absent in the striatal ischemic core (Fig. 2C) compared with the CNPase immunoreactivity in the contralateral striatum (Fig. 2B), indicating substantial loss of myelinating oligodendrocytes. Treatment with rhEPO did not increase ipsilateral CNPase immunoreactivity (28.6±1.8%, n = 4) compared with the saline group (27.7±2.9%, n = 4) 7 days after MCA occlusion. In contrast to the loss of CNPase immunoreactivity, there were many NG2 positive cells in the ischemic boundary region and the SVZ 7 days after MCAo (Fig. 2I-L). Double immunostaining revealed that many NG2 positive cells in these two regions were proliferating cell nuclear antigen (PCNA) positive, a proliferation marker that labels cells in the active cell cycle (Fig. 2I-L), indicating that these NG2 positive cells are within the cell cycle [21], [22]. Animals treated with rhEPO had a significant increase in the number of NG2 positive cells in the peri-infarct corpus callosum and striatum and the SVZ compared with the rats treated with saline (Fig. 2M-P), suggesting that at this stage, EPO amplifies the number of OPCs in both regions.

**Figure 3:**
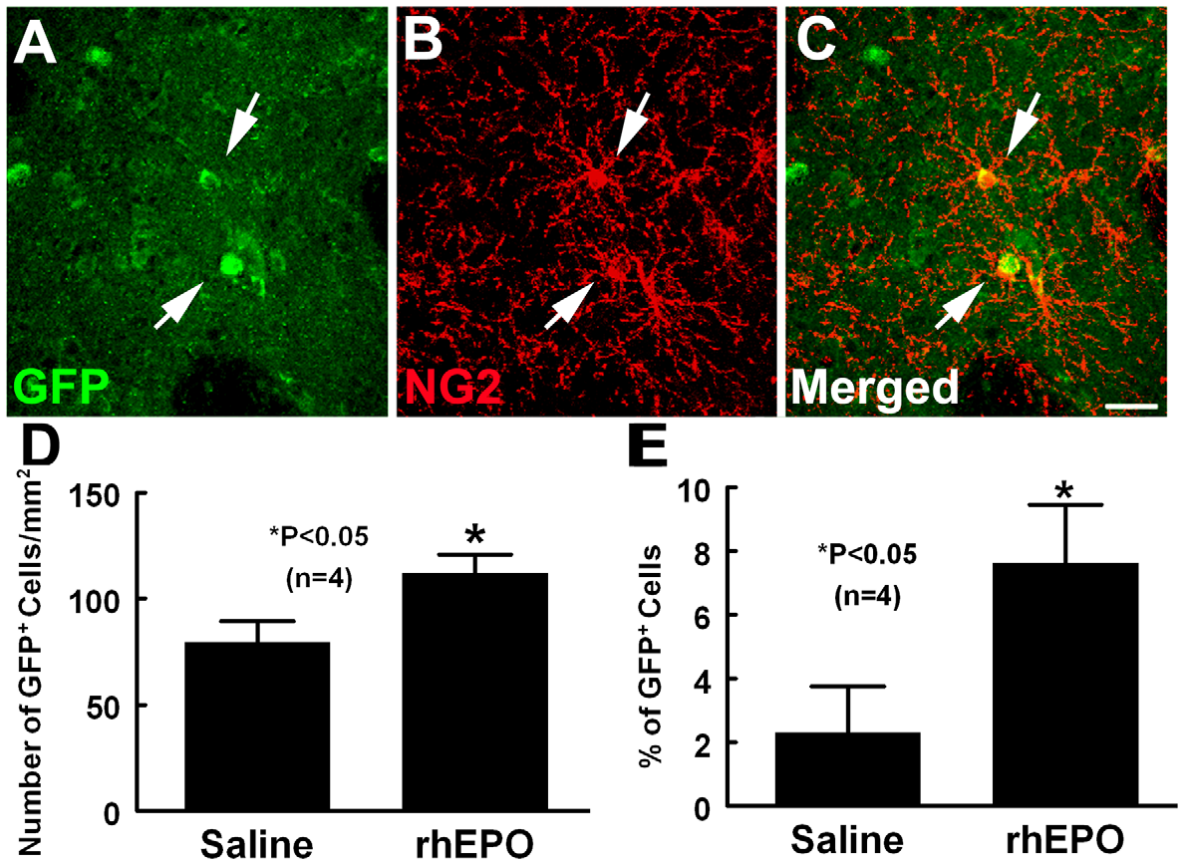
Effects of EPO on the recruitment of OPCs from the SVZ. Panels A to C show GFP (green) and NG2 (red) immunoreactive cells in peri-infarct striatum from a representative rat treated with rhEPO 7 days after stroke. Some of GFP positive cells that were labeled in the SVZ 3 days prior to stroke were NG2 immunoreactive (arrows) in the peri-infarct area. Quantitative data show that the rhEPO treatment significantly increased number of GFP labeled cells (D) and percentage of NG2 positive GFP labeled cells (E) compared with the saline-treated group. *P<0.05 vs saline. Scale bars = 100 µm.

The adult SVZ generates OPCs that disperse throughout the corpus callosum and striatum [8]. To examine whether stroke recruits OPCs from the SVZ, SVZ cells were labeled with the green fluorescent protein (GFP)-lentivirus 3 days prior to stroke, and these rats were sacrificed 7 days after MCAo. In non-ischemic rats, GFP-lentivirus labeled cells were detected within the SVZ one week after injection of the vector (data not shown). However, in the ischemic rats, GFP-positive cells were detected in the peri-infarct regions and some of the GFP-positive cells were NG2 immunoreactive (Fig. 3). Treatment with rhEPO significantly increased the number of GFP and NG2 positive cells in the peri-infarct corpus callosum and striatum compared with rats treated with saline (Fig. 3), suggesting that EPO mobilized OPCs from the SVZ to the peri-infarct corpus callosum and striatum.

**Figure 4:**
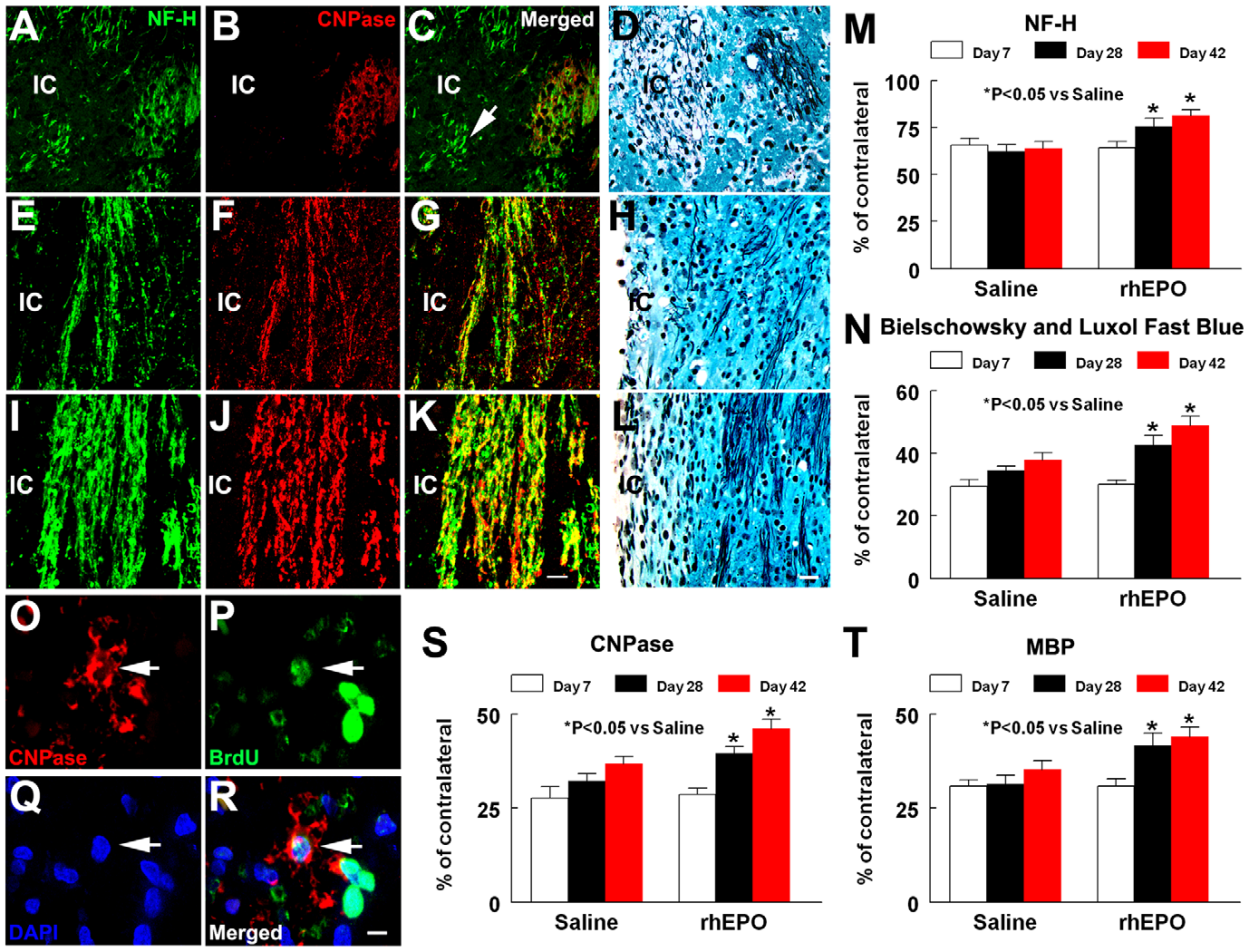
Effects of EPO on axons and myelinating oligodendrocytes. Panels A to L show the double fluorescence immunostaining of NF-H (green, A, C, E, G, I, and K) with CNPase (red, B, C, F, G, J, and K), and Bielschowsky and Luxol fast blue staining (D, H, and L) from representative rats treated with saline (A to D, at 7d; E to H, at 42d) and rhEPO (I to L, at 42d), which were acquired from ipsilateral striatum. An arrow in panels A to C indicate NF-H positive axons with appearances of swellings and bulbs (green) that were not associated with CNPase immumoreactivity (red) 7d after stroke. Quantitative data show that rhEPO treatment significantly increase NF-H immunoreactivity (M) and Bielschowsky and Luxol fast blue (N) positive axons in the peri-infarct striatum compared with saline treated rats 28 and 42d after stroke. Confocal microscopic images (O to R) show that a CNPase immunoreactive cell (red, arrow) was BrdU positive (green, arrow) in the nucleus (DAPI, blue, arrow) of a representative rat treated with rhEPO 28d after stroke. Quantitative data shows that treatment with rhEPO significantly increases the CNPase (S) and MBP (T) immunoreactive area 28 and 42d after stroke. Values are mean ± SE. IC, ischemic core. Scale bars: 50 µm for panels A to L, 20 µm for panels E to H. IC  =  ischemic core. For 7 days, n = 4/saline and n = 4/rhEPO; for 28 days, n = 7/saline and n = 5/rhEPO; for 42 days, n = 7/saline and n = 9/rhEPO.

Twenty-eight and 42 days after MCAo, ischemic rats treated with rhEPO exhibited a significant increase in CNPase and MBP immunoreactivity along the peri-infarct corpus callosum and striatum compared to the ischemic rats treated with saline (Fig. 2 and 4). To examine whether CNPase immunoreactive cells observed 28 days after MCAo are derived from OPCs, we injected bromodeoxyuridine (BrdU) for 7 days starting 1 day after MCAo, which labels proliferating cells during this period, and sacrificed these animals 28 days after ischemia. If proliferating OPCs labeled by BrdU differentiate into myelinating oligodendrocytes during 21 days after BrdU injection, the cells labeled with BrdU will exhibit phenotypes of myelinating oligodendrocytes. Indeed, BrdU and CNPase immunoreactive cells were detected in the ischemic boundary region and confocal images revealed co-localization BrdU in the nucleus and CNPase in the cytoplasm of the same cell (Fig. 4O-R). Some CNPase positive cells exhibited faint BrdU immunoreactivity, suggesting that the OPCs labeled with BrdU continue to divide before differentiation into myelinating oligodendrocytes. These data indicate that new myelinating oligodendrocytes are derived from the OPCs. Quantitative data analysis shows that rhEPO treatment substantially increased the percentage of CNPase cells reactive to BrdU (12.5±2.1%, n = 5) compared with the rats treated with saline (5.3±1.6%, n = 7) 28 days after stroke, suggesting that EPO enhances generation of new myelinating oligodendrocytes.

For both rhEPO treated and saline control rats, the number of NG2 positive cells in the peri-infarct region and the SVZ decreased 28 and 42 days after MCAo compared with the number at 7 days after stroke (Fig. 2). However, ischemic rats treated with rhEPO exhibited a significantly higher number of NG2 positive cells than rats treated with saline 28 and 42 days after stroke (Fig. 2). Quantitative analysis shows that many NG2 positive cells were PCNA positive (Fig. 2), indicating that even 42 days after stroke, OPCs actively proliferate.

### The effect of EPO on axons in the ischemic brain

Our observation that rhEPO increased mature oligodendrocytes prompted us to examine the effect of EPO on myelinated axons. We performed Bielschowsky and Luxol fast blue staining which detects myelinated axons and neurofilament-H (NF-H) immunostaining which labels axons [14]. Seven days after MCAo, NF-H positive axons appearing swollen and bulbous were present in the ischemic striatum and these axons were not surrounded by CNPase immunoreactivity (Fig. 4). Bielschowsky and Luxol fast staininig shows loss of myelinated axons in the ischemic striatum (Fig. 4). These data are consistent with published findings that ischemia directly injures axons [23]. Treatment with rhEPO did not significantly preserve NF-H positive axons compared with the saline treatment 7 days after MCAo (Fig. 4). However, 28 and 42 days after MCAo, ischemic rats treated with rhEPO had a significantly higher density of NF-H immunoreactivity and Bielschowsky and Luxol fast blue positive axons in the striatal ischemic boundary region than ischemic rats treated with saline (Fig. 4). Double immunostaining revealed that CNPase immunoreactive structures were adjacent to NF-H immunoreactive axons in the ischemic boundary region (Fig. 4). These data suggest that EPO enhances myelinated axons.

### The effect of EPO on generation and maturation of OPCs in vitro

**Figure 5:**
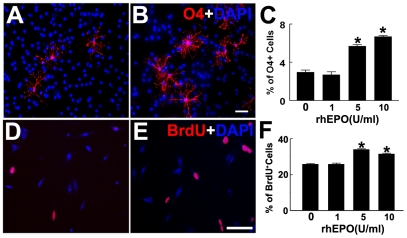
Effects of EPO on generation of OPCs in vitro. Fluorescent microscopic images show O4 (A and B, red) and BrdU (D and E, red) immunoreactive cells in SVZ neural progenitor cells (A and B) and N20.1 cells (D and E), respectively, in the presence (B and E) or absence (A and D) of rhEPO. Panels C and F show quantitative data of O4 positive cells in SVZ neural progenitor cells (C) and BrdU positive cells in N20.1 cells (F) at different concentrations of rhEPO. Blue color represents DAPI positive nuclei. Values are mean ± SE. *P<0.05 vs the control group. Scale bars = 20 µm.

To verify our in vivo findings, we performed in vitro experiments using primary SVZ cells harvested from the adult rat and an oligodendrocyte cell line (N20.1). Adult rodent SVZ cells contain oligodendrocyte progenitor cells that migrate into the white matter [8], [24], [25], [26], [27], [28]. Incubation of SVZ cells with rhEPO significantly increased the number of O4 positive cells in a dose dependent manner with a maximum increase at 10 units/ml (Fig. 5), suggesting that EPO enhances generation of oligodendrocyte progenitor cells.

In addition to the SVZ, many OPCs are present in white matter [29], [30]. We thus incubated immature oligodendrocyte cells (N20.1 cells) with rhEPO and found that rhEPO at concentrations of 5 and 10 units/ml significantly increased the percentage of BrdU immunoreactive N20.1 cells (Fig. 5), indicating that EPO promotes immature oligodendrocyte proliferation.

## Discussion

The present study demonstrates that administration of rhEPO 24 h after embolic MCAo induced sustained OPC proliferation in the peri-infarct white matter and the SVZ. In addition, rhEPO treatment substantially amplified myelinating oligodendrocytes and increased myelinated axons in peri-infarct white matter, which was associated with substantial improvement of functional outcome 6 weeks after stroke. These data suggest that EPO amplifies stroke-induced oligodendrogenesis and axonal remodeling that may contribute to functional recovery after stroke.

Oligodendrocytes are the myelin-forming glial cells in the adult brain [1]. In the current study, the loss of mature oligodendrocytes occurred at the peri-infarct white matter 7 days after MCAo, which is consistent with previous studies showing that the oligodendrocytes are highly susceptible to ischemic insults [2], [3]. Mature oligodendrocytes do not proliferate [9], [10] and new oligodendrocytes are derived from non-myelinating OPCs that are present in the corpus callosum and the striatum [6], [7], [31]. Neural progenitor cells in the SVZ also give rise to nonmyelinating OPCs that disperse throughout the corpus callosum and striatum [8]. Stroke induces generation of OPCs and new oligodendrocytes [5]. However, sources of OPCs and differentiation of OPCs to new oligodendrocytes have not been fully examined. The present study demonstrated that SVZ cells labeled with GFP prior to stroke exhibited a phenotype of OPCs in the peri-infarct region 7 days after stroke. Using BrdU labeling for birth-dating cells, we show that actively proliferating OPCs differentiated into myelinating oligodendrocytes as measured by CNPase positive cells. Collectively, these data suggest that in addition to OPCs in white matter, stroke recruits SVZ neural progenitor cells that contribute to the generation of new oligodendrocytes in peri-infarct white matter, which is consistent with findings that neural progenitor cells originating from the SVZ contribute to white matter oligodendrogenesis after demyelination [8], [32]. More importantly, our in vivo and in vitro data indicate that exogenous EPO substantially amplifies stroke-induced oligodendrogenesis from both sources. However, additional experiments are warranted to examine whether EPO interacts with other mitogenic factors to promote OPC proliferation. A few studies have examined the effect of EPO on oligodendrocytes after cerebral ischemia [16], [17], [33], [34]. Administration of rhEPO immediately after hypoxia-ischemia in the neonatal rat does not protect against white matter damage measured 72 h after hypoxia-ischemia [33]. In a mouse model of cortical injury, administration of rhEPO immediately after injury significantly increased the number of CNPase and MBP positive cells assayed 11 months after the injury [16]. In parallel, the present study shows that treatment with rhEPO did not prevent loss of mature oligodendrocytes but significantly increased the number of OPCs 7 days after stroke, while treatment with rhEPO substantially increased myelinating oligodendrocytes at the ischemic boundary 28 and 42 days after stroke, indicating that EPO likely acts on oligodendrogenesis rather than on protecting oligodendrocytes. Our data are supported by a very recent study showing that delayed administration of rhEPO promotes oligodendrogenesis and improves neurological functional recovery after neonatal hypoxic/ischemic brain injury [17].

Studies in CNPase knockout mice indicate that CNPase expressing cells are required to support axonal integrity [35]. Our data show that EPO-increased CNPase immunoreactivity was closely associated with NF-H positive axons and that EPO augmented myelinated axons measured by Bielschowsky and Luxol fast blue positive axons. Interestingly, using in vivo measurement of diffusion anisotropy which detects axonal tract integrity within white matter after stroke [36], we previously demonstrated that treatment with rhEPO enhanced axonal density around peri-infarct region starting 4 weeks, a time point when a substantial increase in CNPase immunoreactivity was detected in the present study [37]. Thus, we speculate that EPO-amplified oligodendrogenesis facilitates axonal remodeling in the ischemic boundary region, which contributes improvement of functional outcome.

NG2 positive cells are generally considered as OPCs, although NG2 can be induced in activated microglia [5], [38], [39], [40], [41]. NG2 positive cells rapidly proliferate in response to ischemia [5], [38], [39], [40], [41]. In addition to their role as OPCs, NG2 positive cells maintain the extracellular microenvironment of neurons, both in the white and the grey matter [42], and a subpopulation of NG2 positive cells exhibit characteristics of neural progenitor cells [43]. Thus, sustained actively proliferating NG2 positive cells increased by EPO may play additional and as yet unidentified important roles in brain repair after stroke, which warrant further investigation.

## Materials and Methods

All experimental procedures were carried out in accordance with the NIH Guide for the Care and Use of Laboratory Animals and approved by the Institutional Animal Care and Use Committee of Henry Ford Hospital (IACUC approval number: 0811).

### Animal model

Male Wistar rats weighing 350–400 g were subjected to embolic MCA occlusion, as previously described [44].

### Experimental protocols

To test the effect of rhEPO on stroke, rhEPO at a dose of 5,000 units/kg was intraperitoneally administered daily for 7 days starting 24 h after stroke onset (n = 18). As a control group, the same volume of saline was administered to ischemic rats (n = 18) with the identical protocol described above. For mitotic labeling, BrdU (100 mg/kg) was administered daily for 7 days starting 24 h after stroke onset.

Lentiviral labeling: To examine whether SVZ neural progenitor cells migrate to the ischemic boundary region, SVZ cells were labeled with a lentivirus-GFP vector that was constructed, as previously described [45], [46]. Briefly, subconfluent human embryonic kidney 293T cells (ATCC) were cotransfected with 20 µg of a lentiviral vector LV-TH, 15 µg of pCMV-ΔR8.91, and 5 µg of pMD2G-VSVG vectors, After 16 h, the medium was changed, and recombinant lentivirus vectors were harvested 24 h later [46]. For SVZ injection, rats were placed under anesthesia on a Koft stereotaxic apparatus. 5 µl lentivirus-GFP vector was injected into the right SVZ at the coordinates of 1.0 mm lateral to the midline, anterior-posterior at the zero point to the bregma, and 4.5 mm in depth [47] over a 10 min period. The needle was left in the SVZ for an additional 5 min before retraction to avoid reflux. These rats were then subjected to embolic MCA occlusion 3 days after the lentivirus-GFP vector injection. rhEPO at a dose of 5,000 units/kg was intraperitoneally administered daily for 7 days starting 24 h after stroke onset (n = 4). Saline treated rats were served as a control group (n = 4).

### Functional outcome

All functional outcome tests were performed by observers blinded to the treatments 1, 14, and 42 days after onset of MCA occlusion.

### Modified neurological severity score (mNSS)

Rats were tested for motor, sensory, reflex, and balance dysfunctions with the mNSS [48]. Neurological function was graded on a scale of 0 to 18 (normal score, 0; maximal deficit score, 18).

### Foot-fault test

A modified foot-fault test was employed to measure forelimb placement dysfunction [49]. The total number of steps (movement of each forelimb) that the rat used to cross the grid and the total numbers of foot faults for left forelimb were recorded.

### Histopathology and immunohistochemistry

Rats were sacrificed 7, 28 or 42 days after stroke. The brains were removed and consecutive coronal sections at bregma -0.4 to -1.4 mm were prepared. Infarct volume was measured on 7 hematoxylin and eosin (H&E) stained coronal sections using the microcomputer imaging device (MCID) system (Imaging Research, St. Catharines, Ontario, Canada), as previously described [44].

OPCs were identified by staining with antibodies against NG2 (1∶800, Chemicon) and O4 (1∶100, Chemicon). CNPase is a prenylated myelin protein and MBP is an abundant protein component of the myelin sheath, which are highly expressed in mature oligodendrocytes [50], [51]. For evaluation of myelinating oligodendrocytes, an antibody against CNPase (Chemicon) was used at a titer of 1∶200 and an antibody against MBP (Abcam) was used at a titer of 1∶1000. Double immunostaining of CNPase and BrdU (Boehringer Mannheim; 1∶1000) was performed to identify the BrdU incorporating oligodendrocytes. To identify proliferating OPCs, double immunostaining of NG2 and PCNA was performed. Double immunostaining of NF-H (1∶10000; ABR) and CNPase was performed to identify the relationship of axons and oligodendrocytes, respectively. To identify GFP–labeled SVZ cells and their phenotypes, double immunostaining with antibodies against GFP (1∶500, Chemicon) and NG2 (1∶800, Chemicon) was performed on frozen sections obtained from ischemic rats injected with the lentivirus-GFP vector. Double immunolabeling was visualized by secondary antibodies conjugated to FITC and Cy3 (Vector). Bielschowsky and Luxol fast blue staining was also used to detect axons and myelin, respectively [14].

### Image acquisition and quantification

Five coronal sections (8 µm/section) spaced as 100 µm intervals per staining were used from each rat and these coronal sections were within the territory supplied by the MCA at bregma −0.4 to −1.4 mm [44]. Each coronal section was digitized using a 40x objective via MCID system. For quantification of axons and myelinating oligodendrocytes, NF-H, CNPase, MBP, and Bielschowsky and Luxol fast blue positive areas were digitized throughout the peri-infarct corpus callosum and striatum, as well as the contralateral homologous area. Data are presented as the percentage of immunoreactive area at the peri-infarct corpus callosum and striatum compared with the contralateral homologous region on the same section. For quantitative analysis of OPCs, the numbers of NG2 immunoreactive cells were counted throughout the ipsilateral SVZ of the lateral ventricle and peri-infarct area at the corpus callosum and striatum. The number of positive cells for the 5 coronal sections per rat was averaged to obtain a mean number of cells. Data are presented as the density of immunoreactive cells relative to the area of the SVZ and peri-infarct corpus callosum and striatum.

Coronal sections double stained with antibodies against NF-H and CNPase or MBP were imaged using Zeiss confocal microscopy (Zeiss LSM 510 NLO).

### Rat SVZ and mouse N20.1 cell culture

To directly test whether EPO regulates oligodendrogenesis, neural progenitor cells were isolated from the SVZ of adult rats (n = 3), as previously described [52]. To generate neurospheres, SVZ cells were plated at a density of 2×10^4^ cells/ml in the presence of growth medium [52]. The generated neurospheres (primary sphere) were passaged by mechanical dissociation and then plated directly onto laminin-coated glass coverslips in Dulbecco's modified Eagle's (DMEM)-F12 medium containing 2% fetal bovine serum (FBS), but without the growth factors. Passage 1 cells were used in the present study. To examine whether rhEPO enhances oligodendrogenesis, neural progenitor cells were treated with rhEPO at concentrations of 1, 5, and 10 units/ml (epoietinα; Amgen) for 7 days. The cells in the medium without rhEPO were used as a control group. Immunocytochemistry of O4 was performed to identify OPCs. Nuclei were counterstained with 4′, 6′-diamidino-2-phenylindole (DAPI, Vector Laboratories). The number of O4 positive cells and total DAPI cell number were counted and the percentage of O4 positive cells was determined.

To further investigate the effect of EPO on oligodendrocyte cell proliferation, we employed a mouse premature oligodendrocyte cell line (N20.1, generously provided by Dr. Anthony Campagnoni, University of California at Los Angeles), which was obtained from mouse primary cultures of oligodendrocytes conditionally immortalized by transformation with a temperature-sensitive large T-antigen [53]. N20.1 cells grow in DMEM-F12 with 1% FBS and and G418 (100 µg/ml) at 39°C. N20.1 cells were incubated with or without rhEPO at concentrations of 1, 5, and 10 units/ml for 24h, and 20 µg/ml BrdU (Sigma) was added to the cell cultures for 1 h. Immunocytochemistry of BrdU was performed to identify N20.1 cell proliferation. The number of BrdU positive cells and total cell number were calculated by counting 10 random fields in each well with 3 wells per group. The results are presented as a percentage (positive cells divided by total cells).

### Statistics

Data were evaluated for normality. Behavioral data were evaluated with one-way analysis of variance (ANOVA) followed by Student-Newman–Keuls test. Two sample t-tests were used to compare the group difference on histological outcome if data were normal, otherwise nonparametric (Wilcoxon) test was considered. Statistical significance was set at *P*<0.05. All values are presented as mean ± SE.
